# Allergic Rhinitis in Childhood and the New EUFOREA Algorithm

**DOI:** 10.3389/falgy.2021.706589

**Published:** 2021-07-14

**Authors:** Glenis Kathleen Scadding, Peter Kenneth Smith, Michael Blaiss, Graham Roberts, Peter William Hellings, Philippe Gevaert, Marinda Mc Donald, Tania Sih, Suzanne Halken, Petra Ursula Zieglmayer, Peter Schmid-Grendelmeier, Erkka Valovirta, Ruby Pawankar, Ulrich Wahn

**Affiliations:** ^1^Ear, Nose and Throat Department, University College London Hospitals National Health Service Foundation Trust, London, United Kingdom; ^2^Faculty of Medical Sciences, University College London, London, United Kingdom; ^3^Allergy Medical Group, Brisbane, QLD, Australia; ^4^Department of Paediatrics, Medical College of Georgia, Augusta University, Augusta, GA, United States; ^5^National Institute of Health Research Biomedical Research Centre, Southampton General Hospital, Southampton, United Kingdom; ^6^The David Hide Asthma and Allergy Research Centre, Newport, United Kingdom; ^7^Faculty of Medicine, University of Southampton, Southampton, United Kingdom; ^8^Department of Microbiology and Immunology, Department of Otorhinolaryngology, Katholieke Universiteit Leuven, Leuven, Belgium; ^9^Department of Otorhinolaryngology, Academic Medical Center, Amsterdam, Netherlands; ^10^Department of Otorhinolaryngology, Ghent University, Ghent, Belgium; ^11^The Allergy Clinic, Johannesburg, South Africa; ^12^Medical School, University of São Paulo, São Paulo, Brazil; ^13^Paediatric Allergy, University of Southern Denmark, Odense, Denmark; ^14^Karl Landsteiner University of Health Sciences, Krems an der Donau, Austria; ^15^Vienna Challenge Chamber, Vienna, Austria; ^16^Allergy Unit, Dermatology Department, University Hospital of Zurich, Eidgenössische Technische Hochschule Zürich, Zurich, Switzerland; ^17^Department of Lung Diseases and Clinical Immunology, University of Turku and Terveystalo Allergy Clinic, Turku, Finland; ^18^Division of Allergy, Department of Pediatrics, Nippon Medical School, Tokyo, Japan; ^19^Klinik für Pädiatrie m.S. Pneumologie und Immunologie, Charite-Berlin, Berlin, Germany

**Keywords:** pediatric allergic rhinitis, antihistamines, intranasal corticosteroids, fixed dose combinations, allergen specific immunotherapy, asthma, sleep

## Abstract

Allergic rhinitis in childhood has been often missed, mistreated and misunderstood. It has significant comorbidities, adverse effects upon quality of life and educational performance and can progress to asthma or worsen control of existing asthma. Accurate diagnosis and effective treatment are important. The new EUFOREA algorithm provides a succinct but wide- ranging guide to management at all levels, based on previous guidelines with updated evidence and has been adjusted and approved by experts worldwide.

## Introduction

The term rhinitis indicates a symptom complex including two or more of: nasal itching, sneezing, rhinorrhea and nasal blockage. Allergic rhinitis (AR) is IgE-mediated, usually caused by sensitization to inhaled allergens. Other forms of rhinitis are infectious, and non-allergic, non-infectious (1).

**Figure 1:**
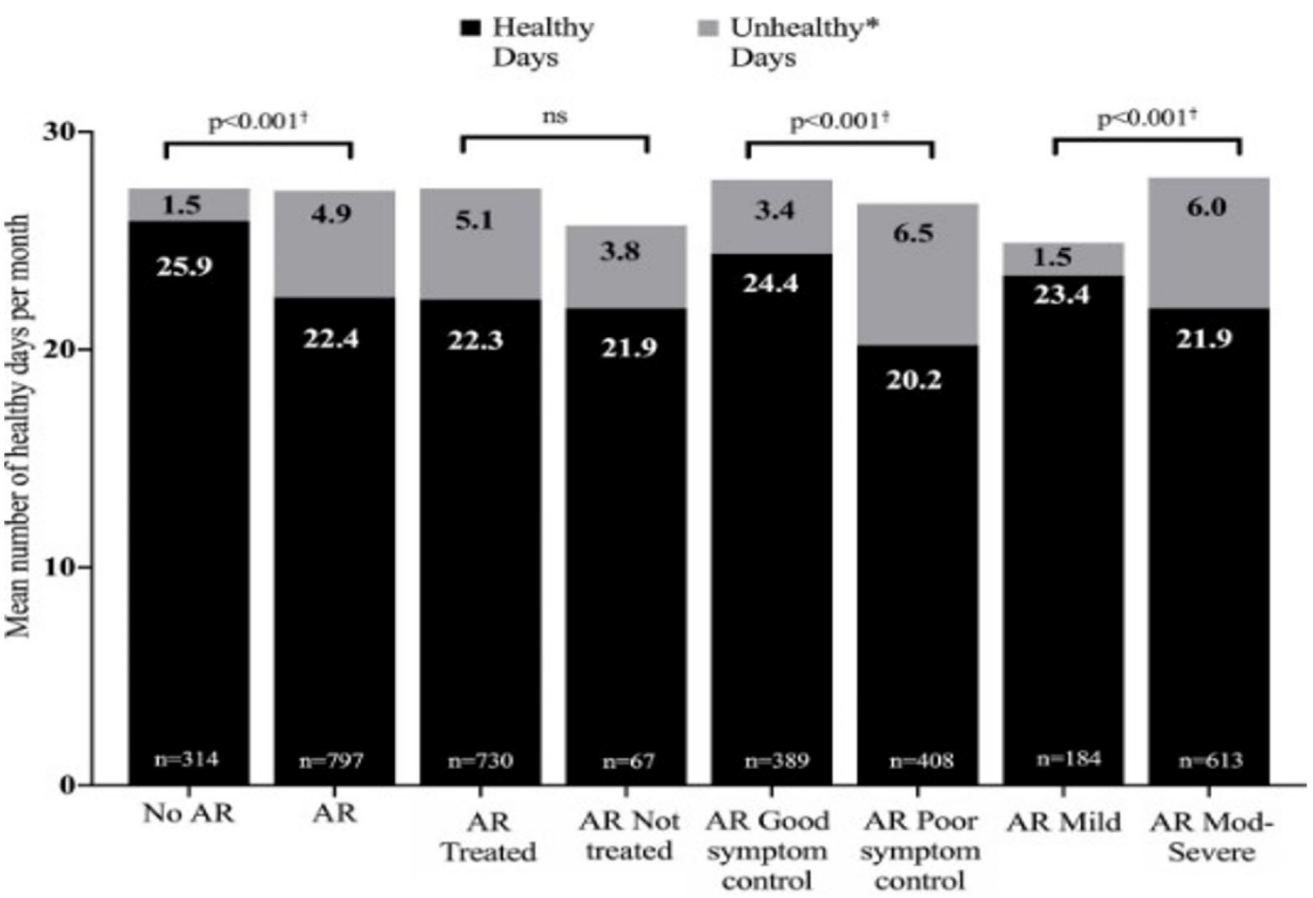
The burden of allergic rhinitis in children aged 6–15 years. Moderate to severe rhinitis and poor symptom control cause over 6 unhealthy days per month. Reproduced from Bosnic-Anticevich S et al. Impact of allergic rhinitis on the day-to-day lives of children: insights from an Australian cross-sectional study. BMJ Open. (2020) 10:e038870. doi: 10.1136/bmjopen-2020-038870, with permission. *Unhealthy days = number of days of poor emotional health and number of days of poor physical health combined. ^†^ Statistically significant difference between groups for each metric (healthy days and unhealthy days).

AR is the commonest immunological disorder in man, with a prevalence of up to 50% in some countries. Often trivialized, in fact it represents a global health problem causing worldwide morbidity. In children AR can not only reduce quality of life via its symptoms, but can affect contiguous organs such as the sinuses, ears and chest and cause sleep problems, leading to reduced school/ work performance (equivalent to that seen in adults), family difficulties and decreased involvement in outdoor activities (2–4). The burden of pediatric AR is shown in Figure 1.

Nasal symptoms and nasal obstruction were more likely to be associated with poor QOL in adolescents than in adults or younger children, respectively (5). In addition AR predisposes to asthma (6), and reduces the control of concurrent childhood asthma, increasing likelihood of hospitalization [OR = 2.34, 95% CI (1.41–3.91)], physician visits (4.4 vs. 3.4, *p* < 0.0001), asthma drug costs [mean GBP 6.7, 95% CI (6.5–7.0)], use of short-acting beta agonists and use of oral corticosteroids (0.091 vs. 0.146, *p* < 0.0001) (7, 8).

The European Forum for Research and Education in Allergy and Airways diseases (EUFOREA) has the mission to implement optimal care for patients suffering from allergies and chronic respiratory conditions (9, 10). Recently, a pocket guide for adult AR was developed by an extended global panel of EUFOREA experts including a novel treatment algorithm (11). The latter has been developed based on existing guidelines and with the aim to allow all care providers to adequately treat adult AR. The need has arisen to develop a pediatric version because the frequency of the common cold and the protean manifestations of AR mean that the diagnosis is often missed, treatment is inadequate and opportunities to alter the course of allergic disease by allergen- specific immunotherapy (AIT) are being wasted.

This article documents the evidence concerning AR prevalence and natural history, then provides management advice with an algorithm based on an update of existing guidelines (12–15). Initially devised by GS and UW, using searches involving the terms “pediatric allergic rhinitis” and “allergic rhinitis in children” and “childhood rhinitis” each meshed with all possible therapies and with “education,” “prevention,” “development,” “outcomes,” “side effects,” and “safety,” this was then altered and adapted by the other authors until a final version was agreed.

## Epidemiology

Most of our current knowledge of pediatric AR epidemiology comes from a widely accepted standardized tool, the International Study of Asthma and Allergies in Childhood (ISAAC) survey, first iterated in 1997 and repeated twice since (16).

In Phase One 156 centers in 56 countries completed the research (17). Prevalence of allergic disease varied more than more than 20-fold between centers (18). Symptoms of rhinitis (and of asthma and eczema) were commoner in some affluent western countries e.g., UK, New Zealand, Australia, but not all, e.g., Spain (16–18). Severe symptoms occurred more frequently in lower and middle income countries, particularly in Africa and Latin America (19, 20), illustrating the important and significant morbidity of rhinitis.

In ISAAC Phase Three two thirds of the centers repeated the study and showed that asthma, rhinitis and eczema symptoms had increased substantially over the previous 15 years, especially in younger children. AR often begins in the under 5 s, but its prevalence increased from 8.5% in individuals aged 6–7 years to 14.6% in those aged 13–14 years (21).

ISAAC Phase Two also provided new information about factors potentially affecting symptom prevalence of asthma, rhinitis, and eczema. Environmental, rather than genetic factors appeared the likely cause of the large variations. Fruit, vegetables, fish and a Mediterranean diet appeared protective; children who ate fast food were more likely to have symptoms (22). A very weak relationship was found between allergy (atopy) and rhinoconjunctivitis, especially in less affluent centers (23). However, such underlying factors may be misunderstood if the phenotype of rhinitis is not diagnosed. The core ISAAC question for diagnosis of AR was: “Has your child ever had a problem with sneezing or a runny or a blocked nose when he/she did not have a cold or the flu?” Subsequent enquiries included itchy eyes and whether an AR or hay fever diagnosis has ever been made, as well as the timing of nasal symptoms. The omission of a detailed history and IgE testing gives a fairly low accuracy for AR diagnosis, estimated as about 60% in a recent Korean study which considered that the ISAAC survey overestimates the true prevalence of AR (24). The roles of the innate and acquired immune systems in rhinitis may differ in individuals, with different allergens and in different parts of the globe; atopy may be more relevant in affluent areas (25).

## The Natural History of Allergic Rhinitis

**Figure 2:**
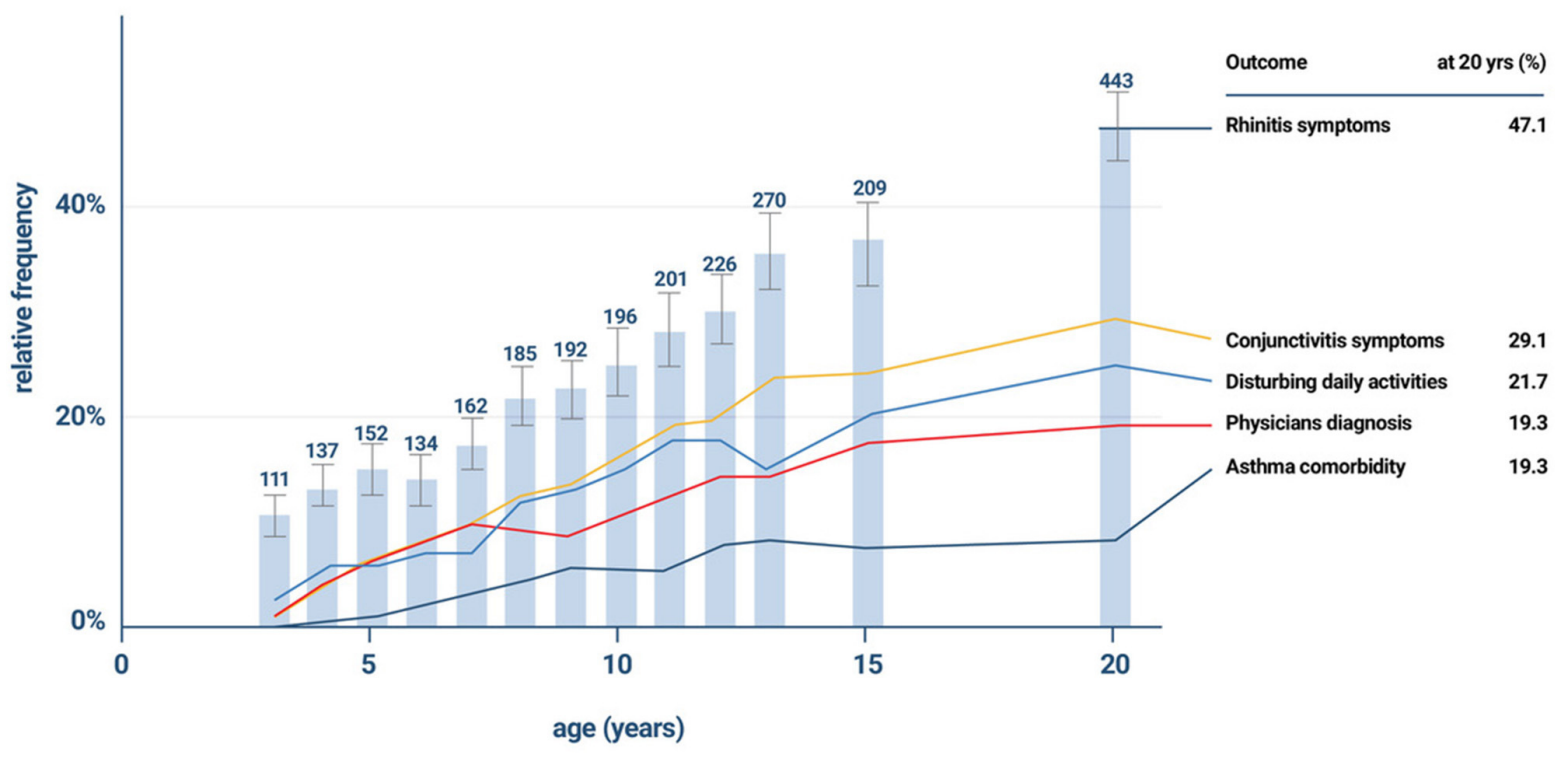
The gradual increase in the incidence of rhinitis symptoms and co- morbidities in the the German Multicentre Allergy Study (MAS), which began in 1990 in five German cities and included 1,314 newborns for the study of the natural course of atopic diseases (26, 27).

**Figure 3:**
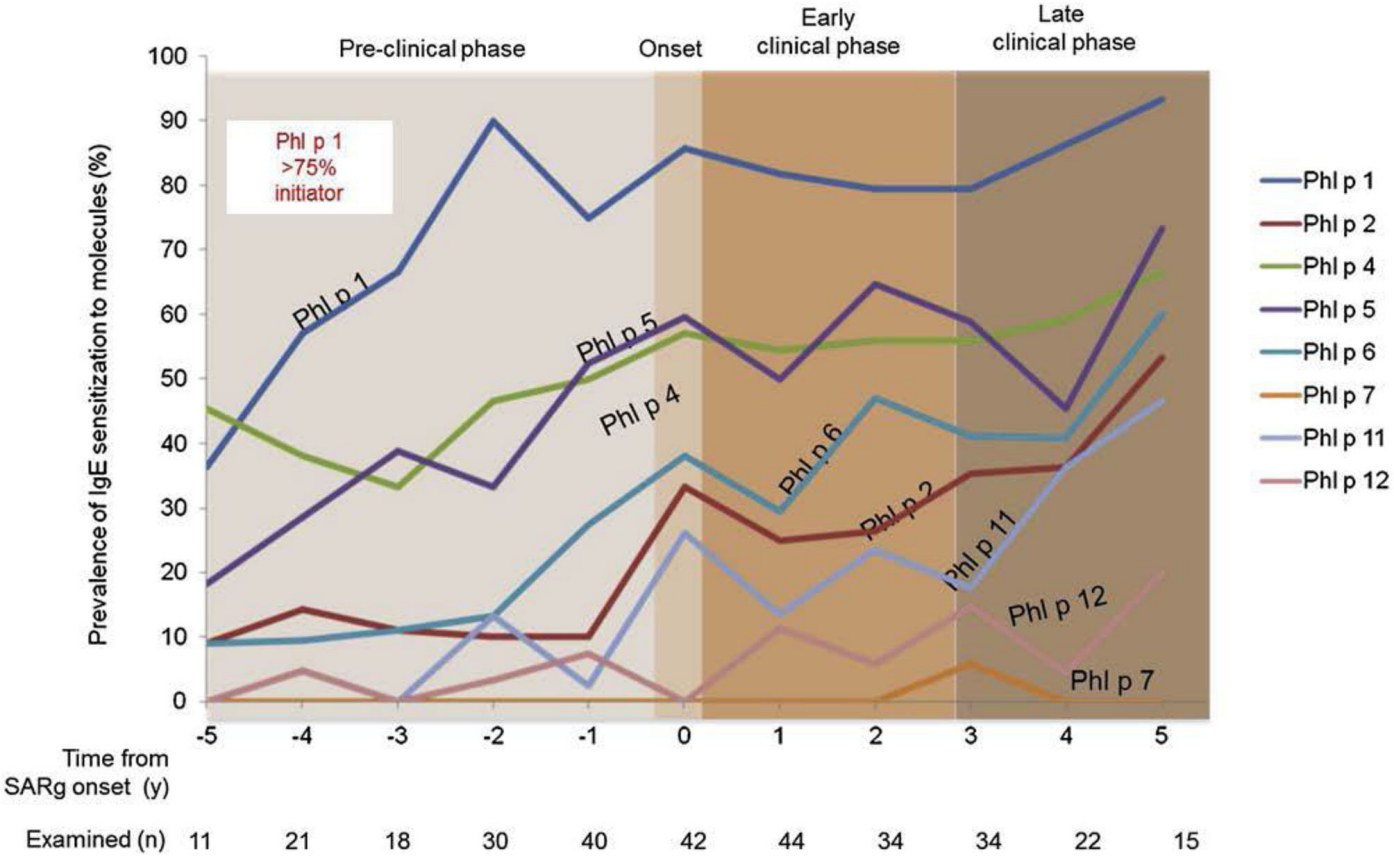
Development of molecular IgE responses of children who developed grass pollen allergy in the first decade of life. Sequential blood samples were obtained in the preclinical phase as well as during the first 5 years with seasonal symptoms. Children were part of the prospective birth cohort study MAS (29).

The best way to understand the natural history of chronic diseases including its major determinants is to observe and prospectively follow cohorts of children for many years, if possible, from birth onwards. Over the last decades a number of birth cohorts have been initiated in Europe and the US. Their main messages regarding allergic rhinitis are as follows:

The incidence of allergic sensitization and allergic (mostly seasonal) rhinitis is very low in the first 2 years. Anecdotal information suggests that very few infants and toddlers develop allergic- type symptoms during any pollen season before the third year of life. In general 2 years (seasons) of environmental allergen exposure seem to be needed before allergic sensitization can be observed by specific serum IgE measurement. The percentage of new cases with seasonal AR increases between the ages of 3 and 12 years at a constant rate of ~2% per year (26, 27). A positive family history (father or mother with allergic rhinitis) is the best predictor of allergic rhinitis (28). Early in life IgE responses to indoor or outdoor allergen sources may only be directed to a minority of allergens, but the 12 month prevalence of sensitization rises from year to year in the first decade of life (Figure 2). A systematic evaluation of the process of sensitization was performed in grass and birch – pollen allergies: The analysis of sequential blood samples for IgE antibodies against grass and birch pollen including individual allergen molecules demonstrated the process of sensitization, which precedes the initiation of symptoms by several years. IgE responses to individual pollen allergens increase with time (molecular allergen spreading), and IgE serum concentrations increase during pre-symptomatic years (Figure 3). Once sensitization to pollen is established, the probability for symptoms within the next 3 years strongly increases (odds ratio 13.6). Simple detection of preclinical allergic sensitization may therefore allow prediction of the onset of hay fever in an allergen-specific manner (29).

Over 60% of children with AR report accompanying eye symptoms, often poorly recognized as allergic in nature (30), by the age of 20 years. Between one and two thirds of them have severe persistent symptoms (according to the ARIA-definition), affecting their daily life. Boys develop rhinitis symptoms earlier, but during adolescence girls catch up and show higher incidences during and after puberty, reaching comparable frequencies by age 20 years (31). This sex shift is most strongly seen in multimorbid patients with both asthma and rhinitis (31).

In atopic children comorbidity is a characteristic feature already in the first 5 years of life. Many children with allergic rhinitis had eczema in infancy. About half of the children with severe persistent allergic rhinitis report wheezing episodes. These findings are in line with the concept of united airways, which suggests that in young children, as in adults, a progression from rhinitis to wheezing can be frequently found and underlies the importance of treating both sites of allergic inflammation to achieve disease control (32).

Rhinitis in childhood is a strong predictor for adolescent- and adult-onset asthma. In the German MAS birth cohort, rhinitis in preschool children was a risk factor for subsequent wheeze when associated with allergic sensitization. This is also true for perennial chronic rhinitis symptoms, which are associated with sensitization to house dust mites. In these cases a causal relationship between allergen exposure and reported symptoms is more difficult to demonstrate than in exclusive seasonal symptoms (33).

## Management of AR in Children

### Diagnosis

#### History

The frequency of common colds in childhood means that AR may be misdiagnosed or ignored. AR is diagnosed by a detailed history, supported by examination of the patient as a whole as well as the nose, plus, if necessary, testing for allergen- specific IgE. The clinical history (see Boxes 1, 2) should note where and when nasal symptoms occur, plus exacerbating and relieving factors. In addition other symptoms, particularly those of asthma, eczema, ENT problems and food allergy should be sought, plus any effects of all these upon sleep and quality of life. A history or a family history of allergic disease and/or immune problems, together with social history, including a review of treatments tried, those currently being taken and their efficacy, should be taken.

Box 1 **Rhinitis symptoms are nasal running, blocking, itching, sneezing, all of which are common in children due to viral colds. This Box gives the clues to an AR diagnosis.**

Rhinitis may be allergic if

• The eyes are involved Itching is noticeable- child gives allergic salute, has allergic crease

• Exposure to a known allergen reliably causes symptoms

• Personal or family history of other allergic diseases

• Some children present with a comorbidity (asthma, atopic eczema, rhinosinusitis, hearing difficulties, sleep disturbance, behavior problems, pollen food syndrome). Always ask about nasal symptoms in such patients

• Always ask about asthma in children with rhinitis and vice-versa.

Box 2 **Red Flags- for specialist attention.**

• Children with unilateral symptoms, severe nasal obstruction +- sleep apnoea

• Children under 2 years and those with a history of rhinitis symptoms present continuously since birth (34, 35)

• Children with nasal polyps

• Those refractory to medical management.

#### Examination

This should include measurement of height, which needs monitoring, especially in children receiving corticosteroids at several sites (36).

**Figure 4:**
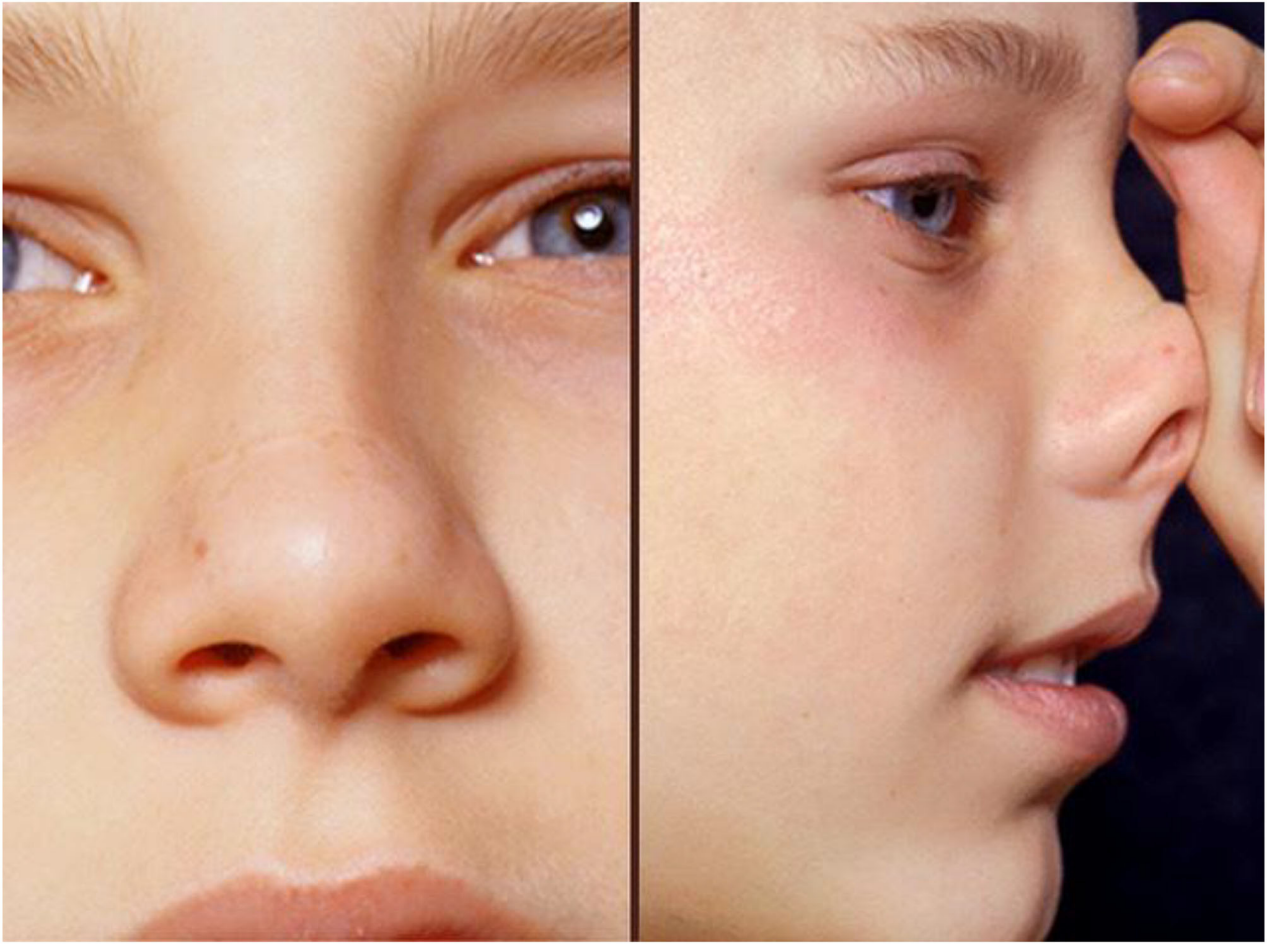
This child shows typical facial changes associated with allergy: he is pale, mouth breathing, with dark circles beneath eyes, a transverse nasal crease, double eye creases and loss of the lateral eyebrow. He is seen giving an allergic salute in the right-hand photo.

The presence of conjunctivitis, nasal allergic crease, allergic salute or double creases beneath the eyes (Dennie–Morgan lines) all suggest that the patient has an allergic diathesis (Figure 4). The ability to breathe through the nose should be tested. In children with moderate to severe AR or uncontrolled symptoms nasal examination is needed, both external and internal. An otoscope will suffice if nasendoscopy is unavailable. Plentiful clear secretions and swollen pale turbinates suggest AR, but the mucosa may be normal or be reddened by INS use. Nasal polyps should prompt testing for cystic fibrosis (37).

ENT referral is advised for patients with bleeding, unilateral disease, high crusting, marked septal deviations and septal perforations as well as those patients who are refractory to medical management (12).

Ear inspection is sensible as otitis media with effusion is a co-morbidity in children with rhinitis, as is auscultation of the chest and an objective measurement of lower airways function, where possible, checking for concomitant asthma and observing the skin for eczema (12).

#### Investigation

Where there is a clear history of symptoms in relation to known allergen exposure a trial of effective treatment, such as intranasal corticosteroids (INS) may be used as a diagnostic tool, with further investigation if unhelpful.

Allergic sensitization can be demonstrated by skin test or specific serum IgE antibody analysis. Both can in principle be applied at any age. If allergen immunotherapy (AIT) is being considered then testing is mandatory. IgE test results need interpretation in the light of the history, as both false- positive and false- negative results can occur.

Skin prick test sensitivity ranges from 68 to 100% and specificity from 70 to 91% (38).

Component- resolved diagnosis, looking at reactivity to specific molecules within an allergen, such as Phl p 1, Phl p 5, Bet v 1 or Pru p 3 is not routinely used, but can predict persistence of AR and the likelihood of future development of asthma or pollen food syndrome. It may also be useful in deciphering cross- sensitization and enabling accurate vaccine content (39–41).

Other tests such as evaluation of nasal nitric oxide and ciliary beat frequency, nasal allergen challenge, CT scans, nasal smears, nasal cultures and analysis of nasal fluid for β-transferrin may be required to include or exclude different forms of rhinitis (37).

### Treatment

Treatments for AR include education, allergen avoidance, pharmacotherapy and AIT (12, 13, 42).

**Figure 5:**
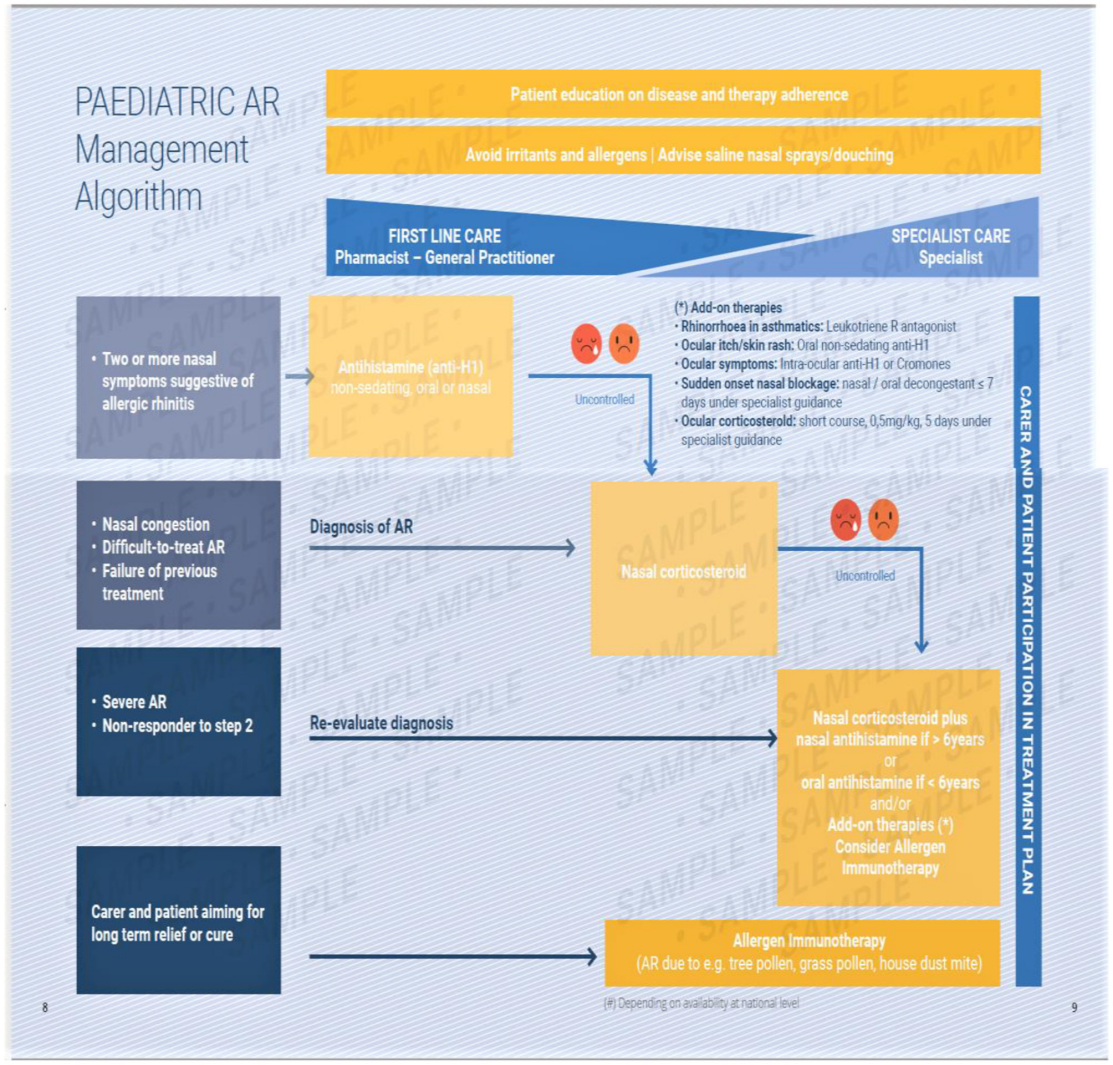
The EUFOREA management algorithm for pediatric allergic rhinitis. This includes measures common to all sufferers and provides a graduated guide to therapy based upon symptoms and their response to therapy. A pictorial visual analog scale is suggested, with poor control being indicated by the two sad faces shown. This requires verification.

The EUFOREA algorithm (Figure 5) includes these and is based upon an update of previous evidence- based guidelines. It covers management of pediatric AR at all levels and of all severities (12–15).

#### Education

**Figure 6:**
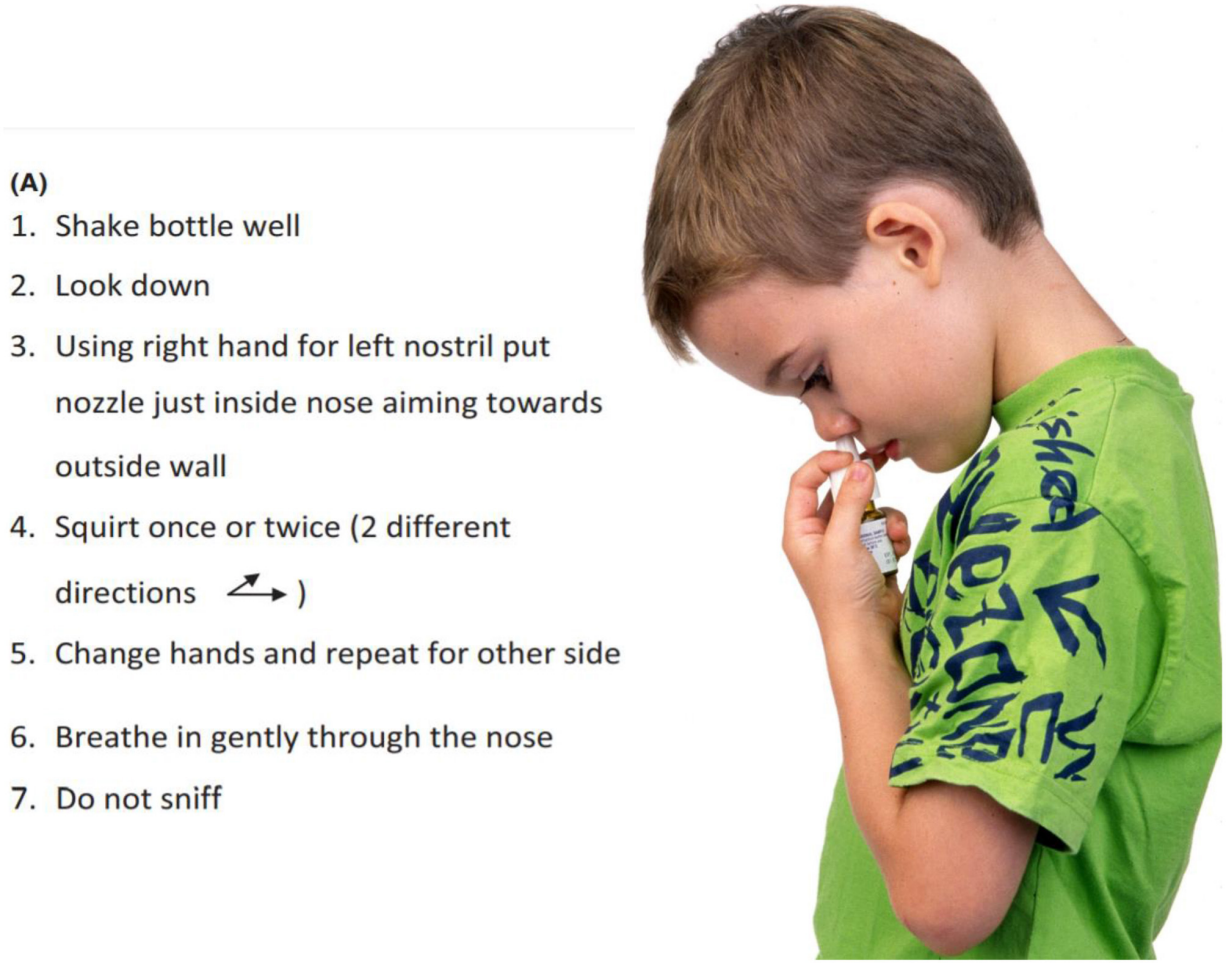
How to use a nasal spray. It is necessary to put the spray onto the lateral walls of the nose, not the septum. It should not be sniffed back hard into the nose but should be moved slowly by mucociliary clearance over the nasal mucosa where the corticosteroid can enter epithelial cells to exert its effects. From Scadding et al. (12), with permission.

Parent/carer education, as well as that of the child, to improve understanding and concordance is vital and also saves time and costs in allergic diseases (12, 43). It includes nature of the disease, finding and eliminating triggers such as allergens and pollutants, explanation of medication suggested and demonstration of the way to use nasal sprays (Figure 6), if prescribed (12). Continuation of patient contact via mobile apps and telehealth may improve outcomes as well as providing data for analysis. If possible children should score their own symptoms, as caregivers usually are less able to adequately capture disease burden (44).

**Figure 7:**
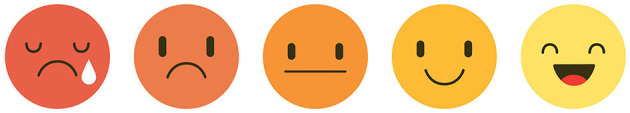
A suggested visual analog scale, using emojis, for younger children to express their feelings about their symptoms.

An emoji visual analog scale is currently under investigation for validity (Figure 7).

#### Allergen/Pollutant Avoidance

In the current COVID pandemic the wearing of face masks is advised for older children. These may also reduce AR symptoms and the possibility of viral spread by sneezing (45).

The UK Royal College of Paediatrics and Child Health (RCPCH) systematic review of 221 studies recently provided evidence linking indoor air pollution to a range of childhood health problems including asthma, wheezing, conjunctivitis, dermatitis, and eczema. Sources of indoor air pollution include smoking, damp, the burning of fossil fuels and wood, dust, chemicals from building materials and furnishings, aerosol sprays and cleaning products. Indoor air quality tends to be poorer in low quality housing where ventilation may be inadequate or insufficient. Improved ventilation and non-allergenic green plants help to mitigate pollution effects^1^.

There is a need for avoidance of allergens and pollutants both inside and outside the home. Persuading parents not to smoke in the home can ease children's symptoms, as can avoidance of gas cookers. Avoidance of major exposures to known allergens, such as pets, house dust mites and mold is sensible, multiple measures do show benefit in AR and asthma, so allergen proof bedding covers and HEPA filters on vacuum cleaners are advised for asthma and AR (https://www.asthma.org.uk/advice/triggers/dust-mites/ and https://www.asthma.org.uk/advice/triggers/indoor-environment/).

The next step is the use of nasal saline which can be universally recommended for all ages. It reduces symptoms and the need for pharmacotherapy and can be used both regularly and/or post-allergen or pollutant exposure. Evidence suggests that seawater or mildly hypertonic saline are most effective (46, 47).

#### Pharmacotherapy

##### Antihistamines

Although widely given as first-line treatment there are problems with pediatric antihistamine use. Firstly there is a paucity of well-controlled studies in AR treatment for some widely available molecules, especially in young children. In particular the first-generation sedating antihistamines lack good evidence of efficacy and are known to have adverse effects such as psychomotor retardation and behavior disturbance and so are not recommended (42).

There is evidence for equivalent efficacy and safety of several second generation antihistamines in pediatric AR. A meta-analysis involving more than 2,500 patients has consolidated the clinical evidence for rupatadine in allergic rhinoconjunctivitis in adults and children (level of evidence Ia, recommendation A). Other recent advances include observational studies of rupatadine in everyday clinical practice situations and approval of a new formulation (1 mg/ml oral solution) for use in children (48). In children aged 6–11, cetirizine, but not loratadine, outperformed placebo (49). However, in a Taiwanese study loratadine outperformed cyproheptadine (50). There is also good evidence for the use of fexofenadine (51, 52), which, together with bilastine (available in Europe for children over 6 years) shows least brain penetration (53).

A further problem is the fact that in drug trials it is often the parent or carer who is scoring the child's symptoms. A scoring system for children to use is needed. We have proposed one using emojis, this is currently being assessed for validity (Figure 7).

Finally antihistamines given orally are only weakly effective in controlling nasal symptoms, so are most suitable for mild AR and where other histamine-mediated symptoms are occurring in the same patient. If one oral antihistamine fails to control symptoms there is no point in trying a different one, the patient should be switched to an intranasal antihistamine or corticosteroid.

Topical intranasal antihistamines act rapidly (15 min) and are more effective than oral ones. Azelastine has shown efficacy and safety in children with AR in 2 European double-blind, placebo-controlled, parallel-group trials and in an open USA study (54). Olopatadine has also shown efficacy in pediatric allergic rhinitis (55). The major adverse effect of intranasal azelastine is a bitter taste, perceived by around 10% of subjects. The taste aversion was less with olopatadine (56).

##### Intranasal Corticosteroids (INS)

Good quality evidence for the efficacy of INS in AR in children exists (12). INS are more effective than H1- antihistamines and leukotriene receptor antagonists, particularly for nasal congestion, although their maximum efficacy requires several hours or days (56). INS are useful first line treatment for AR which is moderate to severe.

The molecules with least systemic bioavailability from the nose are ciclesonide, fluticasone propionate, fluticasone furoate, and mometasone furoate (57). These have good safety data and are preferred for long term pediatric use. Growth in children is a sensitive measure of corticosteroid effects so monitoring it is important.

Teaching correct use of these sprays (Figure 6) reduces common adverse events such as nasal irritation, stinging and epistaxis. Long- term INS use does not damage the nasal mucosa (58).

##### Combination Therapy

For those children whose AR remains uncontrolled despite regular use of an INS the addition of an antihistamine is advised. For those over 6 a fixed dose combination (FDC) nasal spray containing azelastine and fluticasone propionate (MP-Aze-Flu) is available. In a trial this improved quality of life, but not total nasal symptom scores, in all children involved - but did do so in those children who rated their own symptoms, showing the importance of self- assessment (44).

In some countries there is also an FDC with mometasone furoate and olopatadine. These FDCs are rapidly active, more effective than the individual compounds administered alone in over 12 s and adults and are largely well-tolerated (apart from a bitter taste in some patients). FDCs may be most useful in patients, such as teenagers, who tend to treat their symptoms intermittently.

#### Add-On Therapies

##### INS Plus Oral Anti-histamine

In adults combining oral H1- antihistamines and INS does not increase the efficacy of INS, except occasionally for eye symptoms (59, 60). The combination has not been formally tested in children, however addition of an oral antihistamine to an INS makes sense when there are persisting extra-nasal histamine-induced symptoms.

##### Anti-leukotrienes

These have evidence of effectiveness similar to that of oral antihistamines in AR, though there is a spectrum of responsiveness, genetically determined (61, 62).

They may provide useful additional help in children with AR plus asthma, but there should be monitoring for possible adverse psychiatric effects (63).

##### Topical Nasal Decongestants

These cause vasoconstriction and increase the nasal airway but have no effect on other rhinitis symptoms. Regular use can lead to rhinitis medicamentosa. Brief use, under specialist control, is advised when the nose is completely obstructed. This may allow ingress of other therapeutic sprays.

##### Oral Corticosteroids

Specialist prescription of these may be needed when symptoms are extremely severe. Brief use only is necessary because of possible major side effects (12). Injectable depot corticosteroids have an adverse risk profile and should not be used (12).

##### Eye Symptoms

INS reduce eye symptoms to some extent. Cromoglycate or antihistamine eye drops are suitable for patients older than 3 years. Olopatadine is a mast cell stabilizer properties licensed for pediatric use in some countries. Severe eye symptoms warrant an ophthalmological opinion, both to check for vernal conjunctivitis and to enable the use of corticosteroid eye drops which can only be used under such supervision because of the danger of herpetic keratitis (12).

### Allergen Specific Immunotherapy

While avoidance of environmental allergens, unrealistic in many patients, and antiallergic/anti-inflammatory pharmacotherapy are aiming at symptomatic control, allergen specific immunotherapy (AIT), based on the application of relevant allergens to the allergic patients via different routes, is more ambitious. AIT not only reduces symptoms but there is evidence that it can alter the course of disease.

In children as well as in adults this allergen specific treatment has been demonstrated to lead to symptom reduction and less need for medication, not only for the time of treatment, but also for at least 2 years beyond (64).

**Figure 8:**
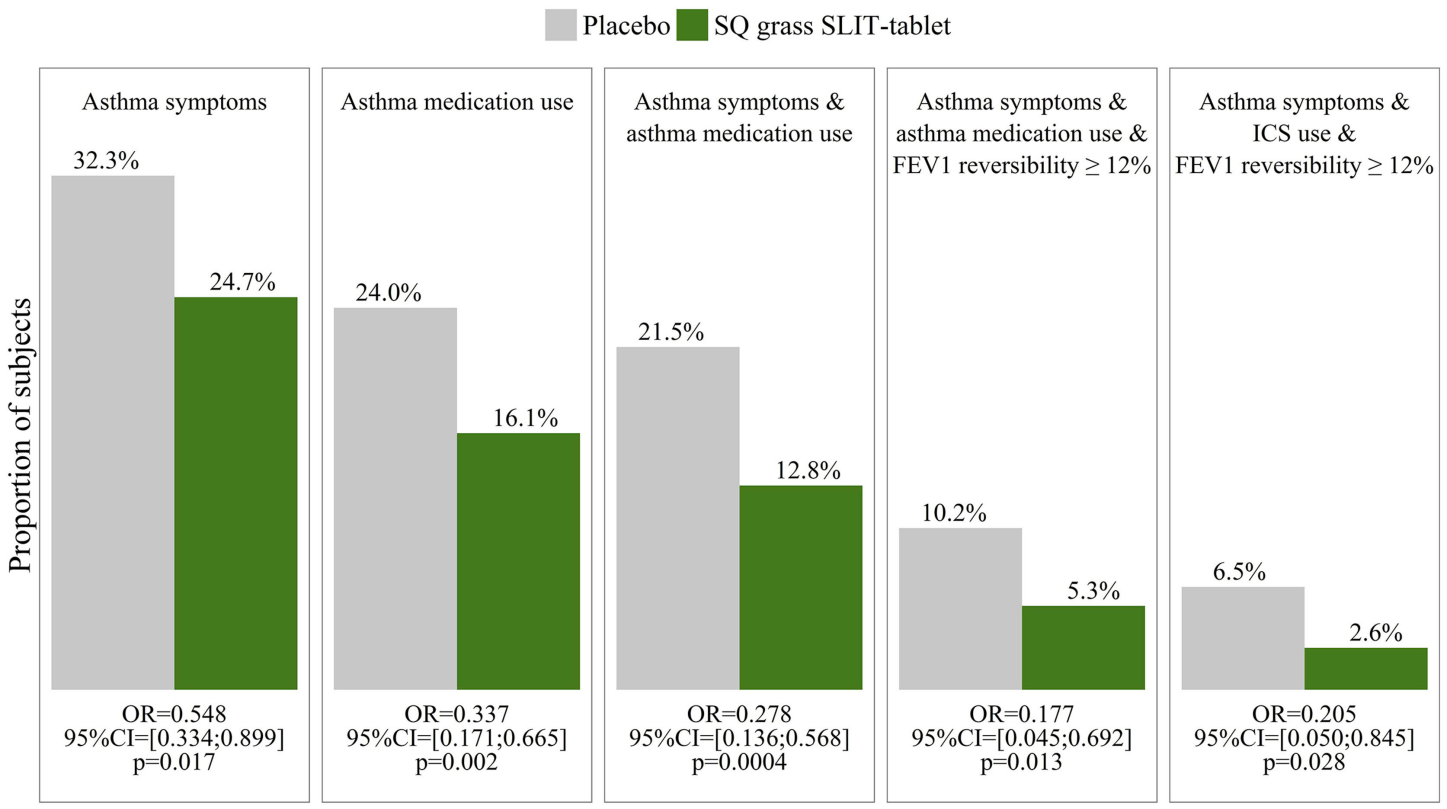
Outcome measures in the GAP study (65) show a reduction in asthma symptoms, medication use and FEV1 reversibility in children with grass pollen allergy who were treated by sublingual immunotherapy (SLIT) to grass pollen, compared to those who received placebo.

Large placebo- controlled trials using subcutaneous and – more recently- sublingual immunotherapy have provided robust evidence for the disease modifying potential of this treatment. In the recent GRAZAX trial which was performed in children with seasonal rhinitis, but no asthma symptoms during the grass pollen season in Europe, it was demonstrated that for a period of 5 years (3 treatment and two follow up years) not only seasonal rhinitis symptoms were reduced, but also the incidence of asthma symptoms as well as the need for asthma medication was reduced for the whole 5 year period (65) (Figure 8).

Immunotherapy had been practiced in Europe and the US for decades without solid scientific evidence for efficacy until Frankland and Augustin (66) published the first placebo-controlled study with grass pollen extracts, and it took until the turn of the century until new criteria for safety and efficacy were defined by health authorities prior to market approval. Nowadays the FDA and the EMA request for all immunotherapy products clinical development plans meeting strict criteria for clinical outcomes such as predefined effect sizes etc. Registration today also includes a pediatric investigational plan. For seasonal pollen allergies both pre- and co-seasonal immunotherapy are widely used; for perennial allergies using allergens from domestic dust (e.g., house dust mites) perennial treatment over 3 years is recommended, in order to achieve fewer symptoms, less need for medication and long term tolerance induction, which lasts for years, even after AIT is discontinued.

Long term safety studies in children indicate that, while sublingual and subcutaneous immunotherapy induce local side effects around the allergen application sites, particularly during the first weeks of treatment, there are very rarely any severe systemic adverse reactions (67). Oral reactions may be reduced by application of the tablet to the vestibulum, between inner lip and teeth, where dendritic cells are more plentiful (67).

Therefore, this treatment can be considered safe from the 5th year of life.

Given the impairment of preschool and school children during daytime activities as well as during sleep, given the increased risk of allergen induced asthma in this age period, it should be recommended to consider allergen specific immunotherapy at the latest after 2 years of allergic symptoms. During recent years the robust clinical effects of SLIT and SCIT in patients with seasonal allergic rhinitis could be confirmed by real world data obtained from data banks in Germany and France (68, 69).

### What About Biologics?

Thus far, no biologic has been marketed for allergic rhinitis. Several studies in children, which combined allergen specific immunotherapy with anti-IgE indicate, that a strong non-specific therapeutic effect of the monoclonal antibody—in addition to the symptomatic effect of AIT—can be observed (70, 71).

In contrast to AIT biologics are significantly more expensive and do not lead to a long-term modification of chronic disease. It seems, however, promising to consider a future treatment with anti-IgE for very severely affected children who showed insufficient response to SIT.

## Prevention

While some preventive interventions seem promising in atopic dermatitis or food allergy (72), the options for allergic rhinitis appear limited. In the German prospective birth cohort study, no single modifiable risk factor was linked to AR. The GINI birth cohort did not observe any reduction of allergic rhinitis after dietary modification in infancy. Multiple other approaches (probiotics) failed in demonstrating preventative effect. Therefore, besides allergen-specific immunotherapy interventions aiming at primary prevention of AR are currently not available. However, high levels of butyrate in early life are associated with a certain degree of protection against atopy (73, 74).

## Discussion

The advent of new treatments and the underuse of current effective therapies such as INS and AIT has meant that a new management guide for pediatric AR became necessary. We have adapted and updated evidence from previous guidelines and combined this with extensive personal experience. Allowing children more control by monitoring their own symptoms and applying their own nasal sprays should improve concordance and control, but this requires confirmation.

## Author Contributions

GS and UW researched the evidence and put together the original document. It was then revised and finally approved by all authors.

## Conflict of Interest

PS was employed by the company Allergy Medical Group (Brisbane). The remaining authors declare that the research was conducted in the absence of any commercial or financial relationships that could be construed as a potential conflict of interest.

## Footnotes

^1^Available online at: https://www.rcpch.ac.uk/resources/inside-story-health-effects-indoor-air-quality-children-young-people.
